# Identification of the B7-H3 Interaction Partners Using a Proximity Labeling Strategy

**DOI:** 10.3390/ijms26041731

**Published:** 2025-02-18

**Authors:** Shujie Liao, Jiamin Huang, Cecylia S. Lupala, Xiangcheng Li, Xuefei Li, Nan Li

**Affiliations:** 1 State Key Laboratory of Quantitative Synthetic Biology, Shenzhen Institute of Synthetic Biology, Shenzhen Institutes of Advanced Technology, Chinese Academy of Sciences, Shenzhen 518055, China; liaosj@siat.ac.cn (S.L.); jiaminhuang1999@163.com (J.H.); cecylia@siat.ac.cn (C.S.L.); 2 University of Chinese Academy of Sciences, Beijing 100049, China; 3 School of Life Science and Technology, ShanghaiTech University, Shanghai 201210, China; lixch2023@shanghaitech.edu.cn; 4 Shanghai Institute for Advanced Immunochemical Studies, ShanghaiTech University, Shanghai 201210, China

**Keywords:** proximity labeling, B7-H3, protein–protein interaction, structure modeling

## Abstract

B7 homolog 3 (B7-H3) has emerged as a promising target for cancer therapy due to its high expression in various types of cancer cells. It not only regulates the activity of immune cells but also modulates the signal transduction and metabolism of cancer cells. However, the specific interaction partners of B7-H3 still remain unclear, limiting a comprehensive understanding of the precise role of B7-H3 in cancer progression. In this study, we report that B7-H3 can bind to resting Raji cells, stimulated THP-1 cells, and even PC3 prostate cancer cells through its IgV domain alone. Furthermore, to identify the potential interaction partners of B7-H3 on these cells, we adopted an ascorbate peroxidase 2 (APEX2)-based proximity labeling strategy, which revealed about 10 key potential interaction partners. Interestingly, our results suggest that CD45 could be a putative receptor for B7-H3 on Raji cells, while the epidermal growth factor receptor (EGFR) could closely interact with B7-H3 on PC3 cells. Based on further computational structure modeling studies, we show that B7-H3 can bind to the epidermal growth factor (EGF) binding pocket of EGFR—surprisingly, with a stronger affinity than EGF itself. Overall, our study provides an effective approach to identifying B7-H3 interaction partners in both immune and cancer cell lines.

## 1. Introduction

B7 homolog 3 (B7-H3), also known as CD276, is a member of the B7 immunoglobulin superfamily [1]. It shares sequence and structural identity with other B7 family members [2,3]. While B7-H3 is expressed at low levels in most normal tissues, it is highly expressed in a wide range of cancers [4]. The expression level of B7-H3 plays a significant role in cancer development. In most cancers, its overexpression is correlated with advanced tumor stage, high tumor grade, and poor prognosis [4,5]. However, in pancreatic cancer, a high expression of B7-H3 is associated with prolonged survival [6]. Therefore, the exact role of B7-H3 in the development of various types of cancer may be complex.

According to the literature, B7-H3 influences cancer development through multiple mechanisms. On the one hand, B7-H3 modulates the activity of various types of immune cells [7]. It has been reported that B7-H3 exerts dual effects, both stimulating and inhibiting T cells [8,9]. Additionally, B7-H3 also interacts with natural killer (NK) cells and monocytes, contributing to an immunosuppressive tumor microenvironment [10,11]. Beyond its immune-modulatory roles, B7-H3 directly supports cancer cell proliferation, migration, and metabolic reprogramming. Studies have shown that B7-H3 enhances the Warburg effect in tumor cells [12]. Moreover, its interaction with key signaling pathways, such as phosphatidylinositol 3-kinase/protein kinase B (PI3K/AKT) and nuclear factor kappa B (NF-κB), promotes epithelial–mesenchymal transition (EMT), angiogenesis, and cancer stemness [13,14,15]. This dual role positions B7-H3 as a unique checkpoint molecule. Emerging therapeutic strategies targeting B7-H3 include monoclonal antibodies, antibody–drug conjugates, and chimeric antigen receptor (CAR) T cells [7]. However, to optimize therapeutic efficacy, a deeper understanding of its interaction partners and regulatory mechanisms is required.

Several studies have proposed interaction partners for B7-H3, but no definitive conclusion has been reached. Hashiguchi et al. identified TREM-like transcript 2 (TLT2) as a counter-receptor of B7-H3 [16]. However, Leitner et al. and Vigdorovich et al. subsequently reported that B7-H3 does not bind to TLT2 [3,9]. Husain et al. and Cao et al. identified interleukin-20 receptor subunit alpha (IL20RA) as a receptor for B7-H3 through in vitro large-scale screening strategies [17,18]. Nevertheless, as the interaction was identified in vitro using bead- or plate-bound libraries of recombinant human single transmembrane proteins, it remains uncertain which cell types B7-H3 interacts with via IL20RA and whether this interaction reflects the actual interactions occurring in living cells.

The ambiguity surrounding B7-H3 interaction partner(s) arises from several challenges. First, B7-H3 appears to exert distinct effects on different cell types [7], suggesting the likely presence of multiple interaction partners. Second, B7-H3 participates in various cell–cell interactions, which are typically transient, dynamic, and spatially restricted, making it technically challenging to identify these interactions using classical protein–protein interaction (PPI) detection strategies [19]. Additionally, interactions occurring at the cell membrane are usually of low abundance, making them easily overshadowed by high-abundance interactions [20]. In this study, we introduce a novel strategy for identifying the interaction partner(s) of B7-H3.

Recently, proximity labeling strategies have demonstrated their utility in studying PPIs [21]. Compared to previous strategies for identifying B7-H3 interaction partner(s), proximity labeling approaches offer several distinct advantages. On the one hand, proximity labeling can be performed in living cells, enabling the identification of endogenous PPIs [21]. On the other hand, these strategies specifically label the proximity proteome of target proteins, significantly narrowing down the list of potential interaction partners compared to labor-intensive screening strategies. Among various proximity labeling catalysts, ascorbate peroxidase 2 (APEX2) enables efficient biotinylation in as little as one minute [22], making it particularly advantageous for detecting weak and transient interactions. Additionally, previous studies have shown that APEX2 exhibits superior specificity compared to horseradish peroxidase (HRP) [23,24].

Leveraging these advantages, we introduce an APEX2-based approach for identifying B7-H3 interaction partner(s). In our approach, a B7-H3–APEX2 fusion protein is purified and incubated with immune and cancer cells. When the B7-H3 domain of the fusion protein interacts with its partners, proteins in proximity are biotinylated by APEX2. The biotinylated proteins, including the putative interaction partner(s), are subsequently enriched and identified by liquid chromatography–tandem mass spectrometry (LC–MS/MS). Using this approach, we identified CD45 as a potential B7-H3 interaction partner on Raji B cells and epidermal growth factor receptor (EGFR) as a potential interaction partner on prostate cancer cell line PC3. Through structural modeling, we propose that B7-H3 may bind to the epidermal growth factor (EGF) pocket of EGFR and subsequently activate the EGFR signaling. These results expand our understanding of the complex role of B7-H3 in cancer development, offering new opportunities for enhancing B7-H3-based cancer therapies.

## 2. Results

### 2.1. B7-H3 Strongly Binds to Resting Raji Cells and Stimulated THP-1 Cells

**Figure 1:**
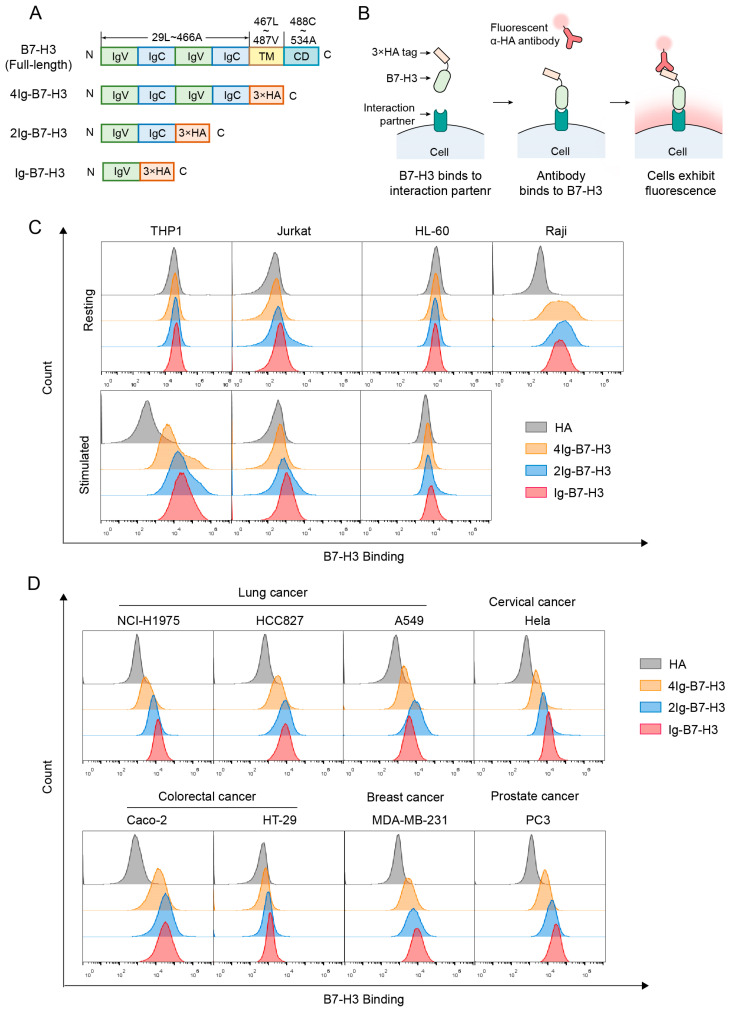
Interaction partners of B7-H3 are widely present across multiple cell types. (**A**) Schematic representation of full-length B7-H3 and its three isoforms. IgV: immunoglobulin-V-like domain; IgC: immunoglobulin-C-like domain; TM: transmembrane domain; CD: cytoplasmic domain. 4Ig-B7-H3: 29Leu~466Ala; 2Ig-B7-H3: 29Leu~238Thr; Ig-B7-H3: 29Leu~139Ala. (**B**) Workflow for evaluating the binding capacity of B7-H3 using flow cytometry. (**C**,**D**) B7-H3 staining of immune and cancer cell lines. Cells were incubated with 15 μg/mL B7-H3 isoforms, with HA peptides at the same concentration were used as a negative control. (**C**) Immune cell lines. (**D**) Cancer cell lines.

The full-length B7-H3 protein comprises tandemly duplicated immunoglobulin-V-like (IgV) and immunoglobulin-C-like (IgC) domains, a transmembrane domain (TM), and a cytoplasmic domain (CD) (Figure 1A). To assess the binding capacity of different B7-H3 isoforms to various cell types, we constructed and purified three distinct B7-H3 isoforms (Figure 1A and Figure S1A). Among these, 4Ig-B7-H3 contains the full extracellular domain of B7-H3, while 2Ig-B7-H3 includes a single set of IgV-IgC domains. Ig-B7-H3 comprises only the IgV domain, which is predicted to serve as the primary interaction domain [3].

Using flow cytometry, we assessed the binding capacity of B7-H3 isoforms to multiple immune cell lines, both before and after stimulation (Figure 1B). Consistent with previous research [11], 4Ig-B7-H3 strongly bound to stimulated THP-1 cells but not to resting THP-1 cells. Furthermore, our results showed that B7-H3 could bind to stimulated THP-1 cells through either the IgV-IgC domains or the IgV domain alone (Figure 1C and Figure S1B).

2Ig-B7-H3 and Ig-B7-H3 weakly bound to resting Jurkat cells, while 4Ig-B7-H3 did not. After stimulation, the binding strength of B7-H3 isoforms to Jurkat cells remained weak (Figure 1C). This observation contrasts with previous findings, which reported that B7-H3 binds to stimulated T cells but not to resting T cells [8]. We attribute this discrepancy to the use of Jurkat cell lines instead of primary T cells in our study.

None of the B7-H3 isoforms bound to resting HL-60 cells. Even after stimulation, the interaction between B7-H3 and HL-60 cells remained minimal (Figure 1C).

Interestingly, all three isoforms of B7-H3, particularly the 2Ig isoform, significantly bound to resting Raji cells (Figure 1C), a finding that has not been reported previously. The mean fluorescence intensity (MFI) of 2Ig-B7-H3-stained Raji cells was more than 80-fold higher than that of the negative control, while 4Ig-B7-H3- and Ig-B7-H3-stained Raji cells exhibited nearly 20-fold higher MFI compared to the negative control (Figure S1B).

Overall, B7-H3 strongly binds to stimulated THP-1 cells and resting Raji cells. The binding strength of three B7-H3 isoforms on the same cell type is nearly identical, demonstrating that B7-H3 is able to interact with its partner using the IgV domain alone.

### 2.2. The Interaction Partner(s) of B7-H3 Are Widely Present on Cancer Cells

To investigate whether B7-H3 isoforms interact with any surface molecules on cancer cells, we incubated B7-H3 isoforms with eight cancer cell lines from five different cancer types, in which a high expression of B7-H3 is correlated with poor prognosis. After incubation, the purified B7-H3 isoforms bound to most of the cancer cell lines (Figure 1D). Results showed that B7-H3 isoforms exhibited strong binding to all assessed lung cancer cell lines, including NCI-H1975, HCC827, and A549 cells (Figure 1D and Figure S1C). Moreover, B7-H3 isoforms also strongly bound to HeLa, MDA-MB-231, and PC3 cells (Figure 1D). However, the binding strength of B7-H3 isoforms varied significantly between the two colorectal cancer cell lines. B7-H3 isoforms strongly bound to Caco-2 cells but weakly interacted with HT-29 cells (Figure 1D).

Taken together, B7-H3 isoforms can bind to various cancer cells, suggesting that the B7-H3 interaction partner(s) are widely present on cancer cells. In addition, B7-H3 can bind to most of the assessed cancer cell lines with the IgV domain alone, suggesting that the IgV domain plays a critical role in mediating interactions between B7-H3 and its interaction partner(s).

### 2.3. An APEX2-Based Proximity Labeling Approach for Identifying B7-H3 Interaction Partner(s)

**Figure 2:**
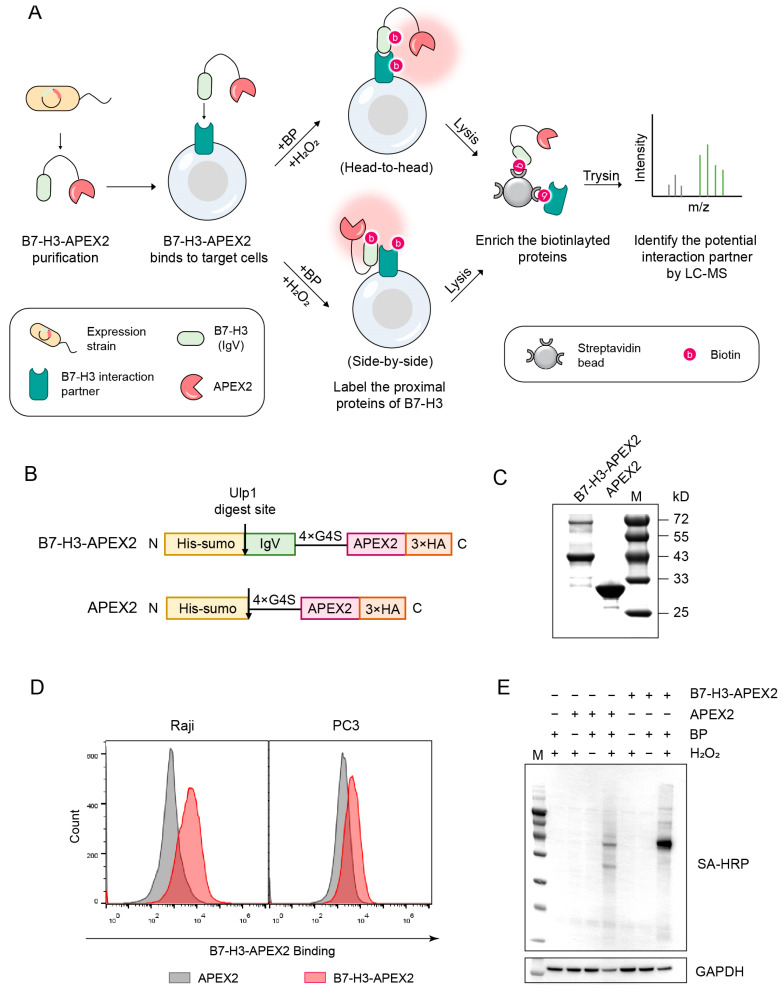
APEX2-based proximity labeling for identification of B7-H3 Interaction partner(s). (**A**) Illustration of APEX2-based proximity labeling to identify B7-H3 interaction partners. (**B**) Expression constructs of B7-H3–APEX2 and APEX2. APEX2, lacking the B7-H3 sequence, served as a negative control. (**C**) SDS-PAGE analysis of purified B7-H3–APEX2 (43.7 kD) and APEX2 (31.8 kD). (**D**) B7-H3–APEX2 and APEX2 staining of Raji and PC3 cell lines. Raji cells were stained with 0.25 µM of each protein, while PC3 cells were stained with 0.5 µM of each protein. (**E**) In vitro labeling activity of B7-H3–APEX2 and APEX2. SA-HRP denotes streptavidin blotting; Glyceraldehyde-3-phosphate dehydrogenase (GAPDH) is shown as a loading control.

We introduced a proximity labeling strategy to identify interaction partner(s) of B7-H3. First, we constructed a fusion protein by fusing APEX2 to the C-terminus of B7-H3 (Figure 2B). The shortest isoform, Ig-B7-H3, was selected for fusion with APEX2, as it retains the interaction ability of B7-H3 while simultaneously reducing the spatial distance between APEX2 and the putative interaction partner(s). A flexible linker (4 × G4S) was inserted between Ig-B7-H3 and APEX2 to improve the accessibility of APEX2 to potential interaction partners. The negative control plasmid, in contrast, encoded only APEX2, without the Ig-B7-H3 interaction domain. Both recombinant proteins were designed with an N-terminal His-SUMO tag.

Rather than directly expressing B7-H3–APEX2 or APEX2 in target mammalian cells, we opted to express and purify these recombinant proteins in the *E. coli* Rosetta (DE3) strain (Figure 2A). Subsequently, the B7-H3–APEX2 or APEX2 proteins were incubated with immune or cancer cells. Through this approach, B7-H3–APEX2 was able to interact with its putative interaction partner in either a head-to-head or side-by-side manner, mimicking interactions between neighboring cells or within the same cell. Once B7-H3–APEX2 bound to the putative interaction partner on the target cells, biotin-phenol (BP) and H_2_O_2_ were introduced to initiate the biotinylation of proteins in close proximity. The biotinylated proteins were subsequently enriched using streptavidin beads and identified by LC–MS/MS. In contrast, the negative control protein, APEX2, could not bind to the putative interaction partner, and thus no biotinylation occurred on the cell membrane.

We assessed the feasibility of the APEX2-based approach. First, we affinity-purified B7-H3–APEX2 and APEX2 proteins using a nickel column (Figure 2C). The His-SUMO tag was then removed from the recombinant proteins via Ulp1 digestion, preventing the affinity tag from interfering with the interaction between B7-H3–APEX2 and the target cells. Subsequently, we evaluated the binding ability of B7-H3–APEX2 to Raji and PC3 cells. The results confirmed that B7-H3–APEX2 retained its ability to bind target cells (Figure 2D). Furthermore, we tested the labeling activity of B7-H3–APEX2 in vitro. The results showed that B7-H3–APEX2 successfully labeled proteins with biotin in a Raji cell lysate following the addition of BP and H_2_O_2_ (Figure 2E). Notably, the labeling efficiency of B7-H3–APEX2 was comparable to that of APEX2 alone, indicating that the fusion with B7-H3 did not compromise the labeling activity of APEX2 (Figure 2E).

### 2.4. CD45 Is a Potential B7-H3 Receptor on Raji Cells

**Figure 3:**
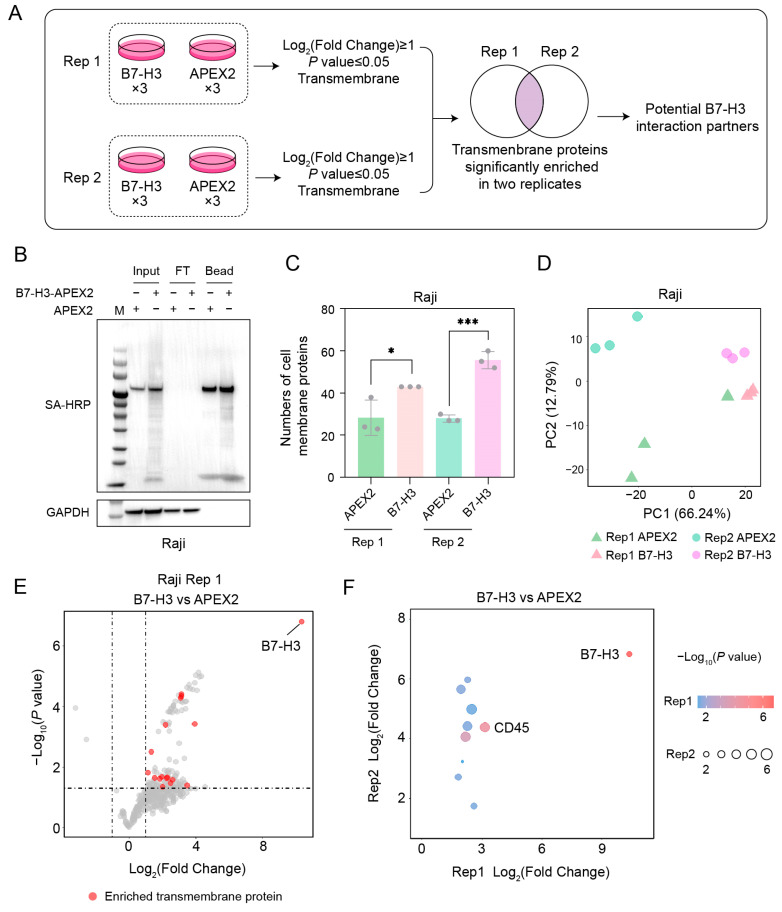
Identification of B7-H3 interaction partners in Raji cells. (**A**) Workflow of proteomics data analysis. The B7-H3 group refers to cells incubated with B7-H3–APEX2, while the APEX2 group refers to cells incubated with APEX2 lacking the B7-H3 sequence. (**B**) Streptavidin enrichment of biotinylated proteins. SA-HRP denotes streptavidin blotting; GAPDH is shown as a loading control. (**C**) Numbers of cell membrane proteins identified in Raji. * means 0.01 < *p*-value < 0.05, *** means 0.001 < *p*-value < 0.005. (**D**) Principal component analysis (PCA) of B7-H3 and APEX2 groups for both replicates. (**E**) Plots of enriched transmembrane proteins (B7-H3/APEX2 fold change ≥ 2 and *p*-value ≤ 0.05) identified in replicate 1 from Raji cells. Red dots represent enriched transmembrane proteins, while gray dots denote unenriched or non-membrane-associated proteins. (**F**) Intersection of enriched transmembrane proteins identified in both replicates.

We initially identified the potential B7-H3 interaction partner(s) on Raji cells, which serve as a model for B cells that play important roles in the anti-tumor immune response [25]. The interaction partner identification workflow is illustrated in Figure 3A. Cells were divided into two groups and incubated with either B7-H3–APEX2 or the negative control protein, APEX2. Each group included three technical replicates. Proximity labeling was then performed in both groups. Subsequently, the biotinylated proteins were enriched and identified using LC–MS/MS. Transmembrane proteins that were significantly enriched in the B7-H3 group were selected. This workflow was repeated twice, and the transmembrane proteins found in the intersection of both biological replicates were manually reviewed and designated as candidates for the B7-H3 interaction partner.

Before performing the proteomic experiment, we assessed the efficiency of proximity labeling and streptavidin enrichment using western blotting (Figure 3B). Biotinylation signals across a wide range of proteins were detected in the cell lysate of the B7-H3 group but were not observed in the APEX2 group, indicating the high specificity of our proximity labeling strategy. After the enrichment, biotinylation signals were still detected in the B7-H3 group. No biotinylation signals were observed in the flow-through, indicating a high efficiency of the enrichment.

Consistent with the western blotting results, we identified and quantified a greater number of cell membrane proteins in the B7-H3 groups compared to the APEX2 groups (Figure 3C). The proteome quantification demonstrated excellent consistency across replicates for the B7-H3 groups (Figure 3D). In contrast, the consistency among replicates in the APEX2 group was lower than that of the B7-H3 group, primarily due to the random nature of the proteins enriched in the APEX2 groups. Through a differential analysis of the two biological replicates, 17 and 39 transmembrane proteins were significantly enriched in the B7-H3 group, respectively (Figure 3E and Figure S2A). The intersection of the two biological replicates yielded nine transmembrane proteins, excluding B7-H3 (Figure 3F). Among these, CD45 was significantly enriched in both biological replicates, suggesting that CD45 is a potential interaction partner of B7-H3.

Given that B7-H3 is minimally expressed in Raji cells, we hypothesized that B7-H3 interacts with CD45 in a head-to-head manner between cancer cells and B cells. To test this hypothesis, we modeled the interaction structure of B7-H3/CD45 and calculated the interaction energy using molecular mechanics generalized Born surface area (MM-GBSA) (Figure S2B). Consistent with our hypothesis, B7-H3 interacts with CD45 in a head-to-head manner, with an interaction energy of −95.543 kcal/mol. The interaction is predicted to involve two IgV domains and one IgC domain of B7-H3 (Figure S2B).

However, CD45 is widely expressed on immune cells (Figure S2C,D), while, in this study, B7-H3 binding was observed only on Raji and stimulated THP-1 cells (Figure 1C). Notably, CD45 is a molecule with multiple isoforms and complex post-translational modifications (PTMs). Different CD45 isoforms are expressed on different immune cells and serve distinct functions [26], which may explain the differential binding of B7-H3 across immune cell types. Furthermore, a previous study reported that another immune checkpoint molecule, BTN3A1, binds to N-mannosylated residues on CD45 [27], suggesting that the various PTMs of CD45 may also contribute to the differential binding of B7-H3 across immune cell types. However, the APEX2-based proximity labeling approach is not capable of distinguishing between isoforms and PTMs. Therefore, further studies are needed to elucidate the detailed interaction mechanism.

### 2.5. Identification of B7-H3 Interaction Partner(s) on PC3 Cells

**Figure 4:**
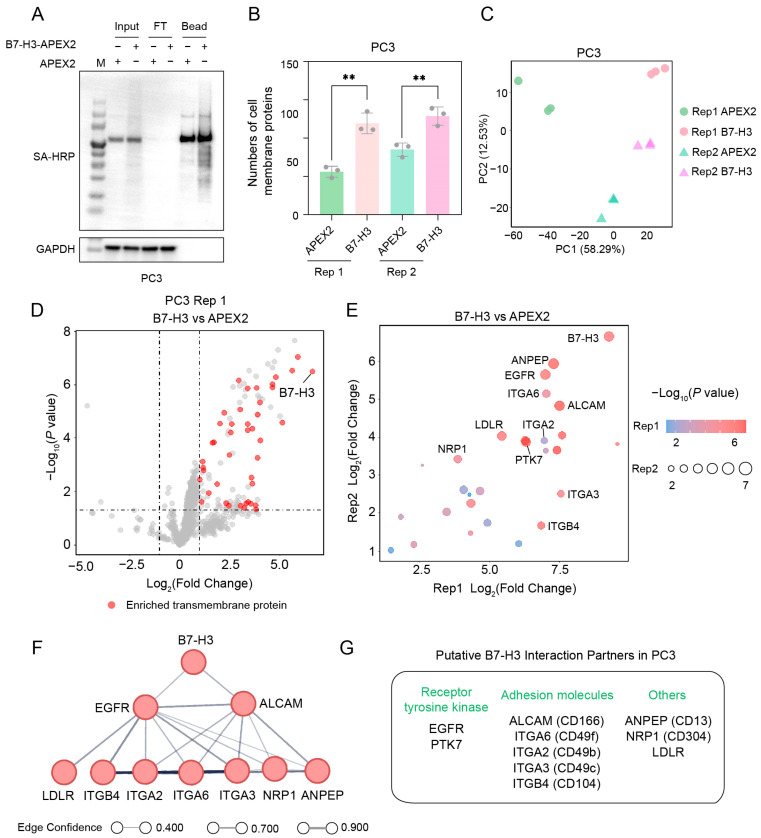
Identification of B7-H3 interaction partners in prostate cancer cells. (**A**) Streptavidin enrichment of biotinylated proteins. SA-HRP denotes streptavidin blotting; GAPDH is shown as a loading control. (**B**) Numbers of cell membrane proteins identified in PC3. ** means 0.005 < *p*-value < 0.01. (**C**) PCA of B7-H3 and APEX2 groups across both replicates. (**D**) Plots of enriched transmembrane proteins (B7-H3/APEX2 fold change ≥ 2 and *p*-value ≤ 0.05) identified in replicate 1 from PC3 cells. Red dots represent enriched transmembrane proteins, while gray dots denote unenriched or non-membrane-associated proteins. (**E**) Intersection of enriched transmembrane proteins identified in both replicates. (**F**) Cell membrane interactome map for B7-H3 in PC3 cells. Interactions were predicted using the STRING database, with edge confidence calculated by STRING. (**G**) Putative B7-H3 interaction partners in PC3 cells. Proteins were categorized according to UniProt function annotations.

We also identified the interaction partner(s) of B7-H3 on a prostate cancer cell line, PC3. The workflow for identifying interaction partner(s) was the same as previously described. Western blotting results showed a good specificity of the proximity labeling and a high efficiency of the streptavidin enrichment (Figure 4A). Through analyzing the proteomic data of PC3 cell line, an average of 119 and 129 cell membrane proteins were quantified from the two biological replicates of the B7-H3 groups, respectively, significantly more than those quantified from the APEX2 groups (Figure 4B). Principal component analysis (PCA) results showed good consistency among the three technical replicates in both B7-H3 groups and APEX2 groups (Figure 4C).

Through the differential analysis of the two biological replicates, 43 and 99 transmembrane proteins were significantly enriched in the B7-H3 group, respectively (Figure 4D and Figure S3A). Excluding B7-H3 itself, the intersection of the two biological replicates yielded 27 transmembrane proteins, 18 of which were located on the cell membrane. From these, we selected the top-10 significantly enriched cell membrane proteins shared by both replicates (Figure 4E). These included two receptor tyrosine kinases, five adhesion molecules, and three other cell membrane proteins (Figure 4G). Interestingly, when we predicted the interactions between B7-H3 and the top-10 proteins using the STRING database, all except protein–tyrosine kinase 7 (PTK7) were clustered into a single interaction network (Figure 4F). B7-H3 was predicted to interact with EGFR and activated leukocyte cell adhesion molecule (ALCAM), which ranked as the top-two and top-four enriched proteins, respectively. Although PTK7 was not involved in the network, we observed a significant positive correlation between the expression level of *B7-H3* and *PTK7* in the Cancer Genome Atlas (TCGA)–prostate adenocarcinoma (PRAD) dataset (Figure S3B), suggesting that PTK7 may also interact with B7-H3.

### 2.6. B7-H3 Is Predicted to Bind to the EGF Binding Pocket of EGFR

**Figure 5:**
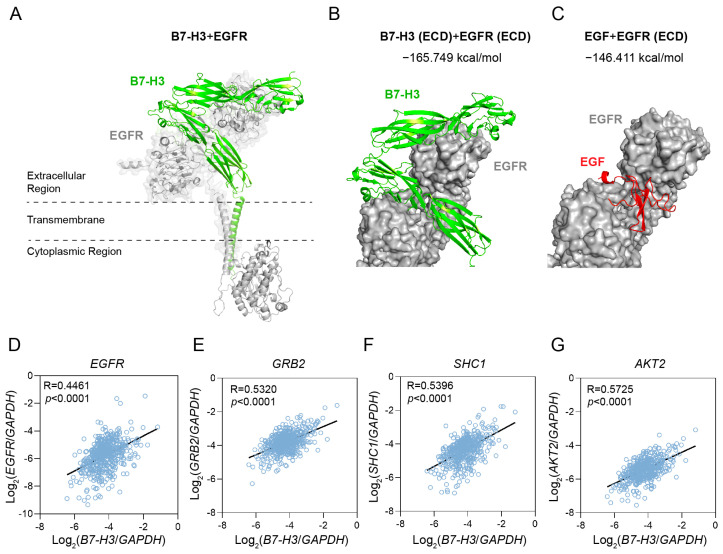
B7-H3 is predicted to bind to the EGF pocket of EGFR. All protein–protein interaction structural models were generated using Alphafold3 and visualized using PyMOL. (**A**) Structure model of the interaction between full-length B7-H3 and EGFR. (**B**) Structural model of the interaction between the extracellular domain (ECD) of B7-H3 and the ECD of EGFR. The interaction energy is −165.749 kcal/mol. (**C**) Structural model of the interaction between EGF and EGFR ECD. The interaction energy is −146.411 kcal/mol. This modeled interaction structure was compared with the experimentally determined structure (PDB ID: 8HGS), showing similar interaction structure and energy (Figure S3C). (**D**–**G**) Correlation analysis of the expression level of *B7-H3* and EGFR signaling molecules in prostate adenocarcinoma (PRAD). RNA transcription data was downloaded from the Cancer Genome Atlas (TCGA).

We modeled the interactions between B7-H3 and EGFR using Alphafold3. The modeled structure shows that B7-H3 interacts with EGFR in a side-by-side manner (Figure 5A). Interestingly, the membrane-proximal IgV and IgC domains of B7-H3 were predicted to bind to the EGF binding pocket of EGFR (Figure 5B). We therefore calculated the interaction energies for the B7-H3/EGFR and EGF/EGFR complexes using Prime MM-GBSA (Figure 5B,C). Surprisingly, the interaction energy for B7-H3/EGFR (−165.749 kcal/mol) was lower than that for EGF/EGFR (−146.411 kcal/mol), suggesting that B7-H3 may exhibit a stronger binding affinity for EGFR.

Notably, ALCAM was reported to bind to the EGF binding pocket of EGFR as an inhibition ligand [28]. Based on this, we hypothesized that B7-H3 might compete with ALCAM for the EGF binding pocket of EGFR, which might explain why ALCAM was identified through B7-H3 proximity labeling. To test the hypothesis, we modeled the interaction structure of ALCAM and EGFR (Figure S3D). The result showed that the interaction energy for ALCAM/EGFR (−141.639 kcal/mol) was higher than that for B7-H3/EGFR, suggesting that B7-H3 possesses a higher binding affinity for EGFR compared to ALCAM.

To investigate the correlation between *B7-H3* and *EGFR* in clinical samples, we analyzed transcriptome data from the TCGA–PRAD dataset. The mRNA expression level of *B7-H3* exhibited a positive correlation with *EGFR* as well as several EGFR downstream signaling molecules (Figure 5D–G), with correlation coefficients ranking in the top 25% of all genes in PRAD. Among these signaling molecules, B7-H3 showed a strong positive correlation with growth factor receptor-bound protein 2 (*GRB2*) (R = 0.5320) and SHC-transforming protein 1 (*SHC1*) (R = 0.5396), both of which directly interact with activated EGFR [29], ranking within the top 10% of all gene correlations in PRAD. Notably, *B7-H3* displayed an even higher correlation with *AKT2*, a key downstream signaling molecule of EGFR, with a correlation coefficient of 0.5725, ranking in the top 5% of all genes in PRAD. These results suggest that B7-H3 might participate in the EGFR signaling pathway. Taken together, B7-H3 is predicted to compete with ALCAM for the EGF binding pocket of EGFR, potentially influencing the regulation of the EGFR signaling pathway.

## 3. Discussion

In this study, we reported that B7-H3 can interact with stimulated THP-1 cells, resting Raji cells and several types of cancer cells. To investigate the molecular basis of these interactions, we introduced an APEX2-based strategy that efficiently identifies B7-H3 interaction partners in both immune and cancer cells. Using this approach, we identified CD45 and EGFR as B7-H3-interacting proteins on Raji cells and PC3 cells, respectively. Structural modeling revealed negative interaction energies for both B7-H3/CD45 and B7-H3/EGFR complexes, indicating strong affinities between B7-H3 and these proteins. Moreover, B7-H3 might bind to the EGF binding pocket of EGFR and participate in the EGFR signaling pathway.

Identifying interaction partners for cell surface immune checkpoint proteins has long been a critical yet challenging task. Numerous studies have proposed various approaches to address this issue. However, these methods suffer from the complex chemical synthesis of photocatalysts [30], extensive gene editing [31,32], or a preference for strong interactions [27]. In contrast, the APEX2-based strategy offers distinct advantages, including the ease of fusing the catalyst to the protein of interest (POI), the elimination of extensive gene editing, and the capacity to detect weak and low-abundance interactions.

Using this APEX2-based proximity labeling strategy, we identified CD45 as a potential counter-receptor of B7-H3 on Raji cells. This finding introduces a novel perspective on modulating B cell activity within the tumor microenvironment. CD45 plays a pivotal role in regulating B cell receptor (BCR) signaling [26], which is essential for antigen presentation and immune activation. Under normal physiological conditions, the B7-H3/CD45 interaction may support immune homeostasis or tolerance. However, in the context of cancer, this interaction might facilitate immune evasion by impairing effective antigen presentation or altering BCR-mediated immune responses.

Therapeutically, disrupting this interaction could restore B cell-mediated immune surveillance, potentially enhancing the efficacy of existing immunotherapies targeting T cells or other immune components. Nevertheless, CD45 is a molecule with multiple isoforms, various PTMs, and complex immune functions. Further studies are required to validate the interaction, clarify the underlying mechanism, and explore the impact of this interaction on tumor immunity.

The identification of EGFR as an interaction partner of B7-H3 in prostate cancer cells reveals a potential direct link between B7-H3 and tumor-promoting signaling pathways. Notably, prior studies have demonstrated that B7-H3 enhances the EGFR–extracellular signal-regulated kinase (ERK) signaling pathway, promoting cancer cell growth, metastasis, and chemoresistance [33]. Moreover, the dual targeting of B7-H3 and EGFR significantly restored chemotherapy sensitivity in colorectal cancer cells both in vitro and in vivo [33]. Consistently, B7-H3 deletion has been shown to reduce the phosphorylation levels of AKT and the signal transducer and activator of transcription 3 (STAT3) while increasing susceptibility to EGFR tyrosine kinase inhibitors in lung adenocarcinoma cells [34].

Based on our findings and previous studies, we hypothesize that B7-H3 binds to the EGF binding pocket, potentially activating EGFR signaling pathways. In cancer, this high-affinity interaction may sustain EGFR activation, promoting aggressive tumor behavior and potentially reducing the efficacy of EGFR-targeted therapies. Combining B7-H3-targeted therapies, such as monoclonal antibodies or CAR-T cells, with EGFR inhibitors may offer a synergistic strategy for overcoming resistance to monotherapies. This approach could be particularly advantageous in prostate cancer, where elevated expression levels of *EGFR* and *B7-H3* are positively correlated and associated with poor clinical outcomes.

This study also has several limitations. From a strategic perspective, fusing the POI with APEX2 may alter the native structure of both proteins, potentially diminishing the binding ability of the POI and the labeling activity of the APEX2. Additionally, the recombinant POI-APEX2 expressed in bacteria lacks the original PTMs, such as glycosylation, which may affect the interaction between POI and its partner. This limitation could be partially addressed by expressing the recombinant protein in mammalian cells, which support more physiologically relevant PTMs. Particularly for this research, interactions were detected in immune and cancer cell lines rather than primary cells. While these cell lines recapitulate certain physiological characteristics of their respective primary cells, notable differences were observed, such as the binding of B7-H3 to Jurkat cells versus primary T cells. This discrepancy suggests the need for the further validation of the findings in primary cells to ensure translational relevance.

## 4. Materials and Methods

### 4.1. Construct Design

The full-length human B7-H3 plasmid was purchased from Genscript, while the *APEX2* plasmid was a gift from Haiyun Gan. All constructs were generated using a pET-28b-SUMO (+) backbone (maintained in our laboratory) through Gibson assembly (New England Biolabs, Ipswich, MA, USA) and subsequently transformed into *E. coli* DH5α strain (*Trans5α* chemically competent cells, TransGen, Beijing, China). Clones containing the desired inserts were verified by DNA sequencing.

### 4.2. Cell Lines and Culture Conditions

THP-1, Jurkat, HL-60, Raji, NCI-H1975, HCC827, Caco-2, and HT-29 cell lines were cultured in RPMI-1640 medium (Gibco, New York, NY, USA) supplemented with 10% Fetal Bovine Serum (FBS) (Gibco), 100 U/mL penicillin, and 100 μg/mL streptomycin (Gibco). A549, Hela, MDA-MB-231, and PC3 cell lines were maintained in DMEM-high glucose (Gibco) with the same supplementation. All cells were incubated at 37 °C in a humidified atmosphere containing 5% CO_2_. Regular mycoplasma testing confirmed that all cell lines were mycoplasma-negative. Details of cell line origins are provided in Table S1.

### 4.3. Immune Cell Stimulation

To stimulate THP-1 cells, phorbol 12-myristate 13-acetate (PMA) (LiankeBio, Hangzhou, China) was added to the culture medium at a final concentration of 100 ng/mL. After 24 h of stimulation, THP-1 cells adhered to the flask surface. At 72 h post-stimulation, the PMA-containing medium was replaced with fresh medium lacking PMA. After an additional 24 h, the stimulated THP-1 cells were dissociated using 0.02% ethylenediaminetetraacetic acid (EDTA) (Beyotime, Shanghai, China) and collected for subsequent experiments.

To stimulate Jurkat cells, 4 μg/mL anti-CD3 and anti-CD28 antibodies (Sino Biological, Beijing, China) in PBS were used to coat a high-binding flask (Corning, Glendale, CA, USA) by incubation at 37 °C for 2 h. After removing the supernatant, Jurkat cells were seeded into the antibody-coated flask. After 12–24 h, the stimulated Jurkat cells adhered to the flask surface. The cells were then dissociated by pipetting and harvested for subsequent experiments.

To stimulate HL-60 cells, 1.3% dimethyl sulfoxide (DMSO) was added to the culture medium. After 60 h of stimulation, the HL-60 cells were harvested for subsequent experiments.

### 4.4. Protein Purification

The *E. coli Rosetta* (DE3) strain (*Transetta* chemically competent cells, TransGen, Beijing, China) was transformed with recombinant protein expression plasmids. A single colony was selected and cultured in LB medium containing 50 μg/mL kanamycin at 37 °C and scaled up to 1 L. When the optical density at OD600 reached approximately 0.6, protein expression was induced with 0.5 mM IPTG at 16 °C for 20 h. Bacterial cells were harvested by centrifugation at 4500× *g* for 15 min, resuspended in 30 mL of lysis buffer (20 mM Tris-HCl, pH 7.5, 350 mM NaCl, 20 mM imidazole, and 10% glycerol), and disrupted using a high-pressure cell disruptor (Antos Nano Technology, Suzhou, China).

The lysate was clarified by centrifugation at 21,000× *g* for 30 min at 4 °C and filtered through a 0.22 μm filter unit (Millipore, Darmstadt, Germany). The supernatant was loaded onto a 5 mL HisTrap High-Performance column (Cytiva, Amersham, UK) using an ÄKTA pure chromatography system (Cytiva). Background proteins were washed with five column volumes of lysis buffer, and the target protein was eluted with a gradient of 5–100% elution buffer (20 mM Tris-HCl, pH 7.5, 350 mM NaCl, 500 mM imidazole, and 10% glycerol) and buffer-exchanged into storage buffer (20 mM Tris-HCl, pH 7.5 and 350 mM NaCl) by centrifugal ultrafiltration.

The SUMO protease Ulp1 was expressed and purified using a similar procedure. The purified Ulp1 was added to the target protein at a 1:100 (mol/mol) ratio and incubated overnight at 4 °C. The digestion mixture was loaded onto a gravity-flow column filled with 2 mL Ni-NTA agarose beads (Cubebio, Monheim, Germany), and the flow-through containing the target protein with the His-SUMO tag removed was collected. The concentration of the purified protein was determined using a NanoDrop spectrophotometer (Thermo Fisher, Waltham, MA, USA).

### 4.5. Flow Cytometry

Next, 1 × 10^6^ cells were harvested and washed once with 3% BSA (*w*/*v*) in PBS. Cells were resuspended in 1 mL of 3% BSA containing 15 μg/mL 3 × HA-tagged B7-H3 isoforms or HA peptides and rotated at 4 °C for 30 min. Subsequently, cells were washed three times with 3% BSA and incubated in 100 μL of 3% BSA containing 2 μg/mL Alexa Fluor 647 anti-HA antibody (Biolegend, San Diego, CA, USA) at 4 °C for 30 min. After incubation, cells were washed three more times with 3% BSA and treated with 10 μM 4′,6-diamidino-2-phenylindole (DAPI) (Biolegend) in 3% BSA for 5 min to exclude dead cells. Finally, cells were washed once and resuspended in 100 μL 3% BSA. The Alexa Fluor 647 intensity was measured using a Cytoflex S flow cytometer (Beckman, Brea, CA, USA). The binding abilities of B7-H3–APEX2 and APEX2 were assessed following a similar procedure.

### 4.6. APEX2 Labeling In Vitro

APEX2 labeling in vitro was conducted following a previously published protocol [23] with slight modification. Total protein from Raji cells was extracted using cell lysis buffer (25 mM HEPES-NaOH, pH 7.5, 1% CHAPS, 1% SDS, 1 mM DTT, 1 mM EDTA, and 1× Roche Complete EDTA-free protease inhibitor) at 4 °C for 15–30 min. The cell lysate was sonicated at 75 W (3 s on, 3 s off) for 3 min, followed by centrifugation at 21,000× *g* for 20 min at 4 °C to remove debris. The protein concentration in the supernatant was measured, and 200 µg of total protein was incubated with 10 μM B7-H3–APEX2 or APEX2 and 0.5 mM biotin-phenol (BP) in 100 µL PBS. Labeling was initiated by adding 1 mM H_2_O_2_, and the reaction was quenched after 1 min with 100 µL of quench buffer (100 mM sodium ascorbate). Negative controls included samples lacking H_2_O_2_, BP, B7-H3–APEX2, or APEX2.

### 4.7. Western Blotting

Protein samples were incubated in 1× SDS loading buffer (YEASEN, Shanghai, China) at 100 °C for 5 min and separated on a premade SDS-PAGE gel (YEASEN). Proteins were then transferred to a PVDF membrane (Millipore, Darmstadt, Germany) using the TS-Blot Transfer System (TSINGKE, Beijing, China). The membrane was blocked with blocking solution (5% BSA in 1 × TBST buffer) at room temperature for 1 h. Following blocking, the membrane was incubated overnight at 4 °C with the appropriate primary antibodies diluted in blocking solution. The primary antibodies used were streptavidin–HRP (Beyotime, A0303, 1:5000) and mouse anti-GAPDH-HRP (Proteintech, Wuhan, China, HRP-60004, 1:5000). After three washes with 1 × TBST buffer (0.1% Tween-20 in TBS buffer), the membrane was treated with SuperSignal West Pico Plus Chemiluminescent Substrate reagent (Thermo Scientific, Waltham, MA, USA) and imaged using the UVP ChemStudio Touch 815 system (Analytik jena, Jena, Germany).

### 4.8. Proximity Labeling In Vivo

A total of 1 × 10^7^ cells were harvested and washed once with 3% BSA (*w/v*) in PBS. The cells were resuspended in 1 mL of 3% BSA containing 0.25 µM B7-H3–APEX2 or APEX2 (0.5 µM for PC3 cells) and rotated at 4 °C for 1 h. Following incubation, the cells were washed three times with 3% BSA and resuspended in 500 µL PBS containing 0.5 mM BP. Proximal labeling was initiated by adding 1 mM H_2_O_2_, and the reaction was quenched after 10 min with 500 µL of quench buffer (100 mM sodium ascorbate). Finally, the cells were washed three times with PBS and either processed immediately or stored at −80 °C for later use.

### 4.9. Pulldown and Sample Preparation for Mass Spectrometry

Cells were lysed in 200 µL of lysis buffer (25 mM HEPES-NaOH, pH 7.5, 1% CHAPS, 1% SDS, 1 mM DTT, 1 mM EDTA, and 1× Roche Complete EDTA-free protease inhibitor) and sonicated at 75 W with 3 s on and 3 s off cycles for 3 min. The lysate was heated at 95 °C for 10 min and subsequently centrifuged at 21,000× *g* for 20 min to remove debris. The protein concentration in the supernatant was measured, and 5% of the supernatant was saved as the input sample for western blotting. Approximately 1.5 mg of protein was diluted to 1 mL with dilution buffer (25 mM HEPES-NaOH, pH 7.5 and 1 × protease inhibitor) and incubated with pre-equilibrated streptavidin sepharose (Cytiva, 17511301) for 2.5–3 h at room temperature (RT) with gentle rotation.

After incubation, the sepharose was centrifuged at 300× *g* for 2 min, and 5% of the supernatant was saved as the flow-through sample for western blotting. The streptavidin sepharose was then washed sequentially as follows: three times with 2× washing buffer (25 mM HEPES-NaOH, pH 7.5, 0.2% CHAPS, 0.2% SDS, 1 mM DTT, and 2 mM EDTA); twice with 1× washing buffer (25 mM HEPES-NaOH, pH 7.5, 0.1% CHAPS, 0.1% SDS, 0.5 mM DTT, and 1 mM EDTA); five times with 150 mM NaCl; five times with 4 M urea in 25 mM HEPES-NaOH, pH 7.5; five times with 30% acetonitrile; and three times with 50 mM triethylammonium bicarbonate (TEAB, Sigma-Aldrich, Darmstadt, Germany). Finally, 5% of the sepharose was saved as the on-bead sample for western blotting.

The remaining sepharose was resuspended in 100 µL 50 mM TEAB containing 10 mM tris (2-carboxyethyl) phosphine hydrochloride (TCEP, Sigma-Aldrich, Darmstadt, Germany) and 40 mM 2-chloro-N-(2,6-dimethylphenyl) acetamide (CAA, Sigma-Aldrich, Darmstadt, Germany). The mixture was incubated for 30 min, followed by three washes with 50 mM TEAB. On-bead digestion was performed by adding 0.5 µg of trypsin (Promega, Madison, WI, USA) in 100 µL of 50 mM TEAB and incubating at 37 °C for 14 h with shaking. The digestion was quenched by adding 0.5% formic acid, and the supernatant was desalted using a C18 StageTip. The desalted peptides were then completely dried in a vacuum centrifugal concentrator (SCIENTZ, Ningbo, China) at 45 °C.

### 4.10. Mass Spectrometry

Peptide samples were dissolved in 0.1% formic acid (*v*/*v*) and separated using a reversed-phase chromatography setup on an Ultimate™ 3000 RSLCnano system (Thermo Fisher Scientific, San Jose, CA, USA) coupled to an Orbitrap Q-Exactive™ HF mass spectrometer (Thermo Fisher Scientific). The separation utilized a trapping column (C18, 20 mm length, 3 µm particle size, Thermo Fisher Scientific, P/N 164535) and an analytical column (C18, 150 mm length, 2 µm particle size, Thermo Fisher Scientific, P/N 164534). Peptides were eluted with a 60 min gradient (Buffer A: 0.1% formic acid in water; Buffer B: 0.1% formic acid in 80% acetonitrile) at a flow rate of 300 nL/min and analyzed in data-dependent acquisition mode.

The Orbitrap Q-Exactive™ HF was operated in positive ion mode with a capillary temperature of 275 °C and a spray voltage of 2.1 kV. Full-scan MS spectra (*m*/*z* 350–2000) were acquired in the Orbitrap with a resolution of 60,000. HCD fragmentation was performed with a normalized collision energy of 28%. The MS2 automatic gain control (AGC) target was set to 5 × 10^4^, with a maximum injection time (MIT) of 50 ms and a dynamic exclusion of 45 s.

### 4.11. Data Analysis

The MS/MS data were searched against the Swiss-Prot database (*Homo sapiens*, downloaded from UniProt) using MaxQuant 2.0.1.0. Searches were conducted with a precursor mass tolerance of 20 ppm and a fragment mass tolerance of 0.5 Da. Enzyme specificity was applied, allowing only tryptic peptides with up to two missed cleavages to be included in the final dataset. Cysteine carbamidomethylation was specified as a static modification, while methionine oxidation and N-terminal acetylation were included as variable modifications. Reverse decoy databases were used in all searches to estimate false discovery rates (FDR). Peptide and protein identifications were quantified and filtered to ensure an FDR of less than 1%.

Search results were filtered to exclude contaminants, reverse decoys, proteins identified only by site, and proteins with fewer than two unique peptides. Proteins with fewer than three LFQ intensities in both groups were also excluded. Missing values were imputed with the minimum value. Differential analysis identified proteins with a fold change ≥ 2 and *p*-value ≤ 0.05 as enriched in B7-H3 groups. Among these, cell membrane proteins (as annotated in the UniProt subcellular location database) enriched in both replicates were selected as putative B7-H3 interaction partners.

Potential interactions between these proteins were predicted using the STRING database (www.string-db.org, accessed on 16 October 2024). CD45 transcriptional data and protein expression data of different cell lines were obtained from the Human Protein Atlas (www.proteinatlas.org, accessed on 26 November 2024). Transcriptional data of PRAD clinical samples were obtained from TCGA database (portal.gdc.cancer.gov, accessed on 14 December 2024) and normalized by the GAPDH expression to eliminate differences between samples.

### 4.12. Structure Modeling

For the protein structure modeling, all the protein sequences were obtained from the UniProt website (www.uniprot.org, accessed on 14 November 2024), while the high-confidence protein–protein interaction structural models were modeled using Alphafold3 (alphafoldserver.com, accessed on 14 November 2024) [35]. For the EGFR complex with EGF, the modeled structure was compared with experimentally solved structure (PDB ID: 8HGS). The interaction energy for all PPIs were calculated using the Prime MM-GBSA module (Version 2.5.5, Schrödinger, New York, NY, USA). In each case, the top-score model from Alphafold3 was used to calculate MM-GBSA interaction energies. In each PPI involving B7-H3, the B7-H3 structure was treated as the ligand and the interaction partner as the receptor. Prime MM-GBSA uses OPLS-AA force field and VSGB 2.0 implicit solvation model to estimate the binding energy of the receptor–ligand complex. The binding energy was calculated as $\Delta G\left(bind\right)=E_{Complex}-\left(E_{Ligand}+E_{Receptor}\right)$. All resultant structural figures were generated using PyMOL software (Version 2.5.5, Schrödinger, New York, NY, USA).

The brand, product number, and origin of reagents used in this study are listed in Table S3.

## 5. Conclusions

In conclusion, we introduced an APEX2-based proximity strategy to identify interaction partners of cell membrane immune checkpoint proteins. This strategy is compatible with primary cells and efficient to detect weak interactions, either in a head-to-head or side-by-side manner. Using this approach, we identified CD45 as a potential head-to-head interaction partner of B7-H3 and EGFR as a potential side-by-side interaction partner. Our study provides new insights into identifying novel interaction partners on plasma membranes both intercellularly and intracellularly. Furthermore, the potential interaction partners identified in our study shed new light on the role of B7-H3 in the cancer progression.

## Data Availability

The MS proteomics data have been deposited to the ProteomeXchange Consortium [36] via the iProX [37,38] partner repository (ProteomeXchange: PXD059197).

## Supplementary Materials

The following supporting information can be downloaded at: https://www.mdpi.com/article/10.3390/ijms26041731/s1.
