# Supplementary material for: Identification of the B7-H3 Interaction Partners Using a Proximity Labeling Strategy
Source: Int J Mol Sci. 2025 Feb 18;26(4):1731. doi: 10.3390/ijms26041731 (PMC11855656; doi:10.3390/ijms26041731)
Supplement: Supplementary file 1 [file ijms-26-01731-s001.zip › 1_Supplementary figures.pdf]

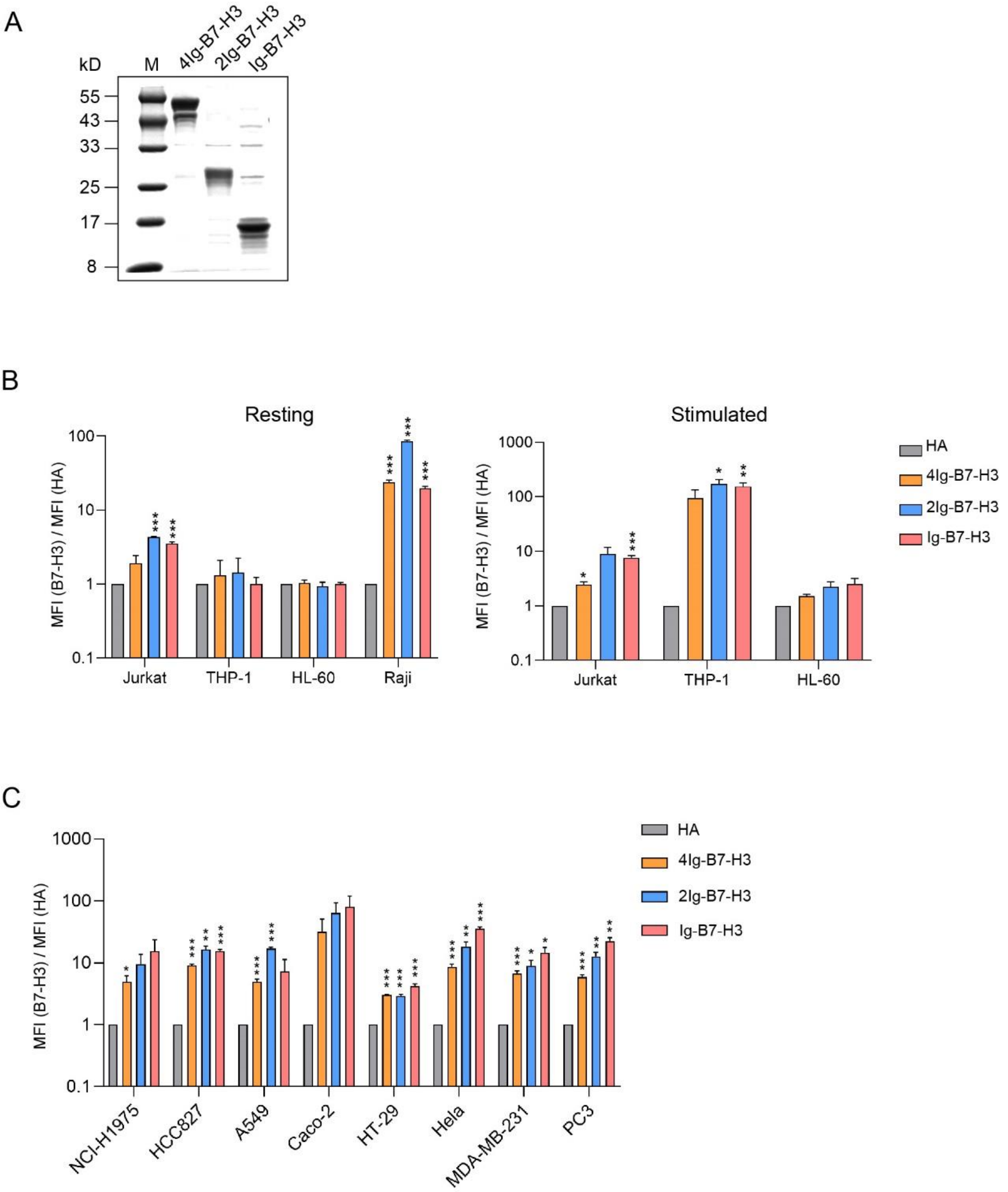

Supplementary Figure S1 Quantification of the binding ability of B7-H3 to different cell types. (A) SDS-PAGE analysis of purified B7-H3 isoforms following His-SUMO tag removal. Molecular weights: 4Ig-B7-H3, 50.5 kDa; 2Ig-B7-H3, 26.0 kDa; Ig-B7-H3, 15.3 kDa. (B, C) The binding ability of B7-H3 to different cell types was characterized by the ratio of MFI (B7-H3) to MFI (HA). A t-test was performed to compare the B7-H3 groups with the HA group. MFI, Mean Fluorescence Intensity. (B) Quantification of the binding ability of B7-H3 to resting and stimulated immune cells. (C) Quantification of the binding ability of B7-H3 to different cancer cells.

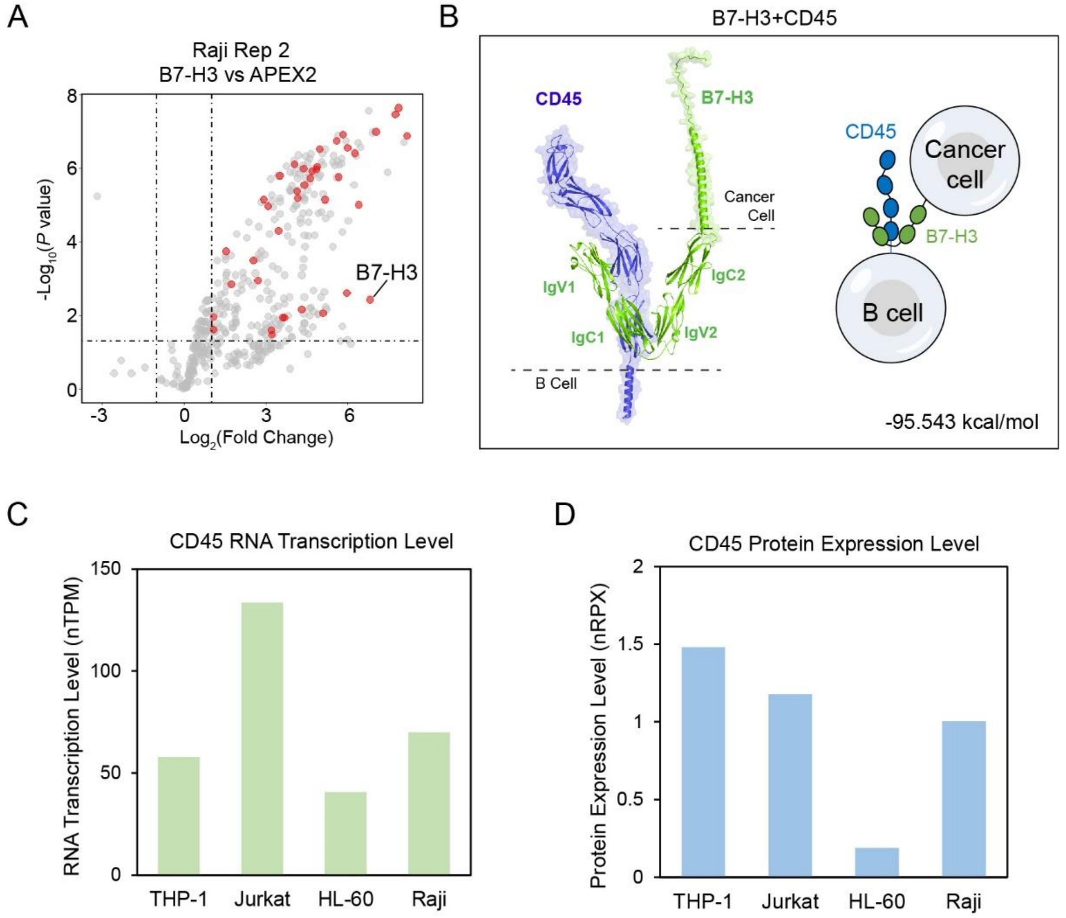

Supplementary Figure S2 CD45 is a potential B7-H3 receptor on Raji cells. (A) Plots of enriched transmembrane proteins in replicate 2 of Raji cells. Cutoff: B7-H3/APEX2 fold change ≥2 and *P*-value ≤0.05. Red dots, enriched transmembrane proteins; Gray dots, proteins not enriched or not associated with membranes. (B) B7-H3 interact with CD45 in a head-to-head manner with an interaction energy of -95.543 kcal/mol. The PPI was modeled using Alphafold3. (C) Normalized transcript per million (nTPM) values for CD45, based on RNA data from the Human Protein Atlas database. (D) Normalized relative protein expression (nRPX) values for CD45, derived from the MS proteomics dataset of the Pan-Cancer Atlas project.

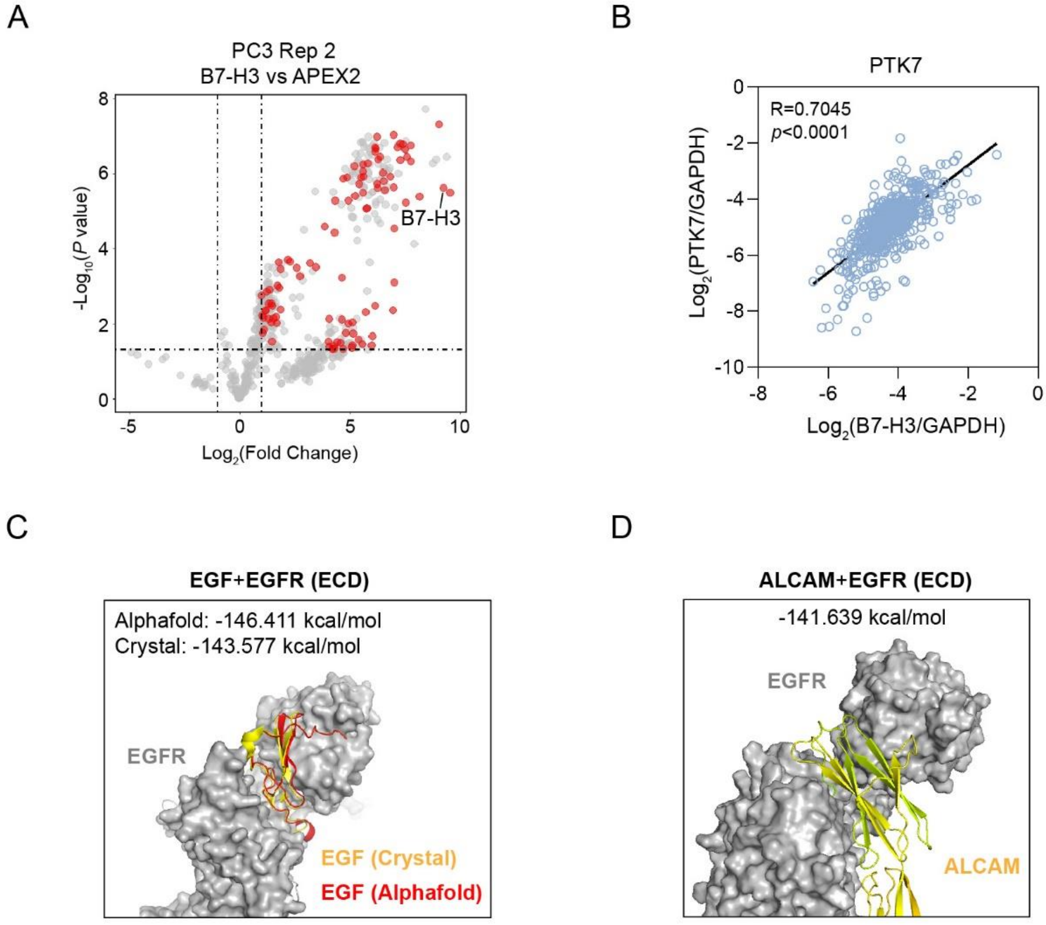

Supplementary Figure S3 EGFR and ALCAM are the potential B7-H3 interaction partners on PC3 cells. (A) Plots of enriched transmembrane proteins in replicate 2 of PC3 cells. Cutoff: B7-H3/APEX2 fold change ≥2 and *P*-value ≤0.05. Red dots, enriched transmembrane proteins; Gray dots, proteins not enriched or not associated with membranes. (B) Correlation analysis of the expression level of B7-H3 and PTK7 in PRAD. RNA transcription data was downloaded from TCGA. (C) Modeled structure of EGF/EGFR superposed with crystal structure of the same (PDB ID: 8HGS). Interaction energy of the Alphafold structure is -146.411 kcal/mol; Interaction energy of the crystal structure is -143.577 kcal/mol. (D) Structural model of the interaction between ALCAM and EGFR ECD. The interaction energy is -141.639 kcal/mol.
