# Supplementary material for: Identification of the B7-H3 Interaction Partners Using a Proximity Labeling Strategy
Source: Int J Mol Sci. 2025 Feb 18;26(4):1731. doi: 10.3390/ijms26041731 (PMC11855656; doi:10.3390/ijms26041731)
Supplement: Supplementary file 1 [file ijms-26-01731-s001.zip › 2_Supplementary tables.pdf]

Supplementary Table S1. Sources of cell lines

Cell type	Source
THP-1	Preserved by our lab
Jurkat	Purchased from Procell, CL-0129, China
HL-60	A gift from Chenli Liu Lab
Raji	Purchased from Procell, CL-0189, China
NCI-H1975	A gift from Xianen Zhang Lab
HCC827	A gift from Xianen Zhang Lab
A549	Preserved by our lab
Hela	Preserved by our lab
Caco-2	A gift from Shuqiang Huang Lab
HT-29	A gift from Shuqiang Huang Lab
MDA-MB-231	Preserved by our lab
PC3	Purchased from Procell, CL-0185, China

Supplementary Table S2. Antibody list

Antibody	Source
Human CD3 Antibody-Human IgG4 (GMP-grade)	Sino Biological, GMP-10977-H001, China
Human CD28 Antibody-Human IgG2	Sino Biological, 11524-H001, China
Alexa Fluor® 647 anti-HA.11 Epitope Tag Antibody	Biolegend, 682404, USA
Streptavidin–HRP	Beyotime, A0303, China
HRP-conjugated GAPDH Monoclonal antibody	Proteintech, HRP-60004, China

Supplementary Table S3. Regent list

**Regent**	**Source**
NEBuilder® HiFi DNA Assembly Master Mix	NEB, E2621L, USA
Trans5α chemically competent cells	TransGen, CD201-01, China
Transetta chemically competent cells	TransGen, CD801-02, China
RPMI-1640 medium	Gibco, 11875119, USA
DMEM-high glucose	Gibco, 11054001, USA
Fetal Bovine Serum (FBS)	Gibco, A5670701, USA
Penicillin-streptomycin	Gibco, 15140122, USA
Phorbol 12-myristate 13-acetate (PMA)	LiankeBio, CS0001, China
0.02% ethylenediaminetetraacetic acid (EDTA)	Beyotime, C0198, China
High-binding flask	Corning, 3290, USA
Dimethyl sulfoxide (DMSO)	Sigma-Aldrich, D2650, Germany
HisTrap High Performance column	Cytiva, 17524701, UK
Ni-NTA agarose beads	Cube Biotech, 31103, Germany
4',6-Diamidino-2-Phenylindole (DAPI)	Biolegend, 422801, USA
SDS loading buffer	YEASEN, 20315ES, China
SDS-PAGE gel	YEASEN, 36247ES, China
PVDF membrane	Millipore Sigma, ISEQ00010, Germany
TS-Blot Transfer System	TSINGKE, TSP8211, China
SuperSignal West Pico Plus Chemiluminescent Substrate reagent	Invitrogen, 34580, USA
Complete EDTA-free protease inhibitor	Roche, 04693132001, Switzerland
Streptavidin sepharose	Cytiva, 17511301, UK
Triethylammonium bicarbonate (TEAB)	Sigma-Aldrich, 18597, Germany
tris (2-carboxyethyl) phosphine hydrochloride (TCEP)	Sigma-Aldrich, C4706, Germany
2-chloro-N-(2,6-dimethylphenyl) acetamide (CAA)	Sigma-Aldrich, C0267, Germany
Trypsin Gold, Mass Spectrometry Grade	Promega, V5280, USA
Empore™ SPE C18 Disks	Sigma-Aldrich, 66883-U, Germany
